# Hydrogen Sulfide: Emerging Role in Bladder, Kidney, and Prostate Malignancies

**DOI:** 10.1155/2019/2360945

**Published:** 2019-11-03

**Authors:** Masoud Akbari, Emrullah Sogutdelen, Smriti Juriasingani, Alp Sener

**Affiliations:** ^1^Department of Microbiology & Immunology, Schulich School of Medicine & Dentistry, University of Western Ontario, Dental Sciences Building, Rm 3014, London, Ontario, Canada N6A 5C1; ^2^Matthew Mailing Center for Translational Transplant Studies, University Hospital, London Health Sciences Center, 339 Windemere Road, London, Ontario, Canada N6A 5A5; ^3^Department of Surgery, Schulich School of Medicine & Dentistry, St. Joseph's Health Care London, PO BOX 5777, STN B, London, Ontario, Canada N6A 4V2; ^4^Multi-Organ Transplant Program, University Hospital, London Health Sciences Centre, 339 Windemere Road, London, Ontario, Canada N6A 5A5

## Abstract

Hydrogen sulfide (H_2_S) is the latest member of the gasotransmitter family and known to play essential roles in cancer pathophysiology. H_2_S is produced endogenously and can be administered exogenously. Recent studies showed that H_2_S in cancers has both pro- and antitumor roles. Understanding the difference in the expression and localization of tissue-specific H_2_S-producing enzymes in healthy and cancer tissues allows us to develop tools for cancer diagnosis and treatment. Urological malignancies are some of the most common cancers in both men and women, and their early detection is vital since advanced cancers are recurrent, metastatic, and often resistant to treatment. This review summarizes the roles of H_2_S in cancer and looks at current studies investigating H_2_S activity and expression of H_2_S-producing enzymes in urinary cancers. We specifically focused on urothelial carcinoma, renal cell carcinoma, and prostate cancer, as they form the majority of newly diagnosed urinary cancers. Recent studies show that besides the physiological activity of H_2_S in cancer cells, there are patterns between the development and prognosis of urinary cancers and the expression of H_2_S-producing enzymes and indirectly the H_2_S levels. Though controversial and not completely understood, studying the expression of H_2_S-producing enzymes in cancer tissue may represent an avenue for novel diagnostic and therapeutic strategies for addressing urological malignancies.

## 1. Hydrogen Sulfide

**Figure 1:**
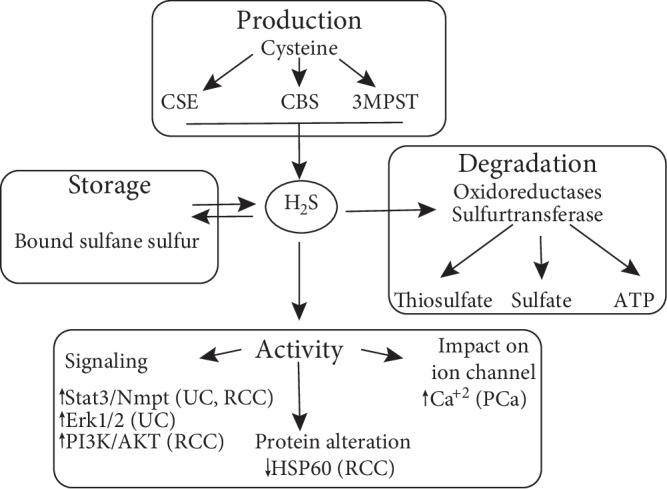
Synthesis, storage, degradation, and activity of H_2_S, especially in urinary cancers. H_2_S has roles in different pathways of urinary cancers such as signaling or ion channel. Abbreviations: CBS: cystathionine *β*-synthase, CSE: cystathionine *γ*-lyase, 3-MPST: 3-mercaptopyruvate sulfurtransferase, Stat3/Nmpt: signal transducer and activator of transcription 3/nicotinamide phosphoribosyltransferase, HSP60: heat shock protein 60, PI3K/AKT: phosphatidylinositol 3-kinase, UC: urothelial carcinoma, RCC: renal cell cancer, PCa: prostate cancer.

For several centuries, hydrogen sulfide (H_2_S) was known as a pollutant, but now its physiological and pathophysiological processes are well known. H_2_S is widely recognized as the third endogenous gasotransmitter after carbon monoxide (CO) and nitric oxide (NO) in mammals and some other species, with similar pathophysiological characteristics [1, 2]. H_2_S is synthesized endogenously by reverse transsulfidation and oxidation of cysteine [3–6], by three tissue-specific enzymes: cystathionine *β*-synthase (CBS), cystathionine *γ*-lyase (CSE), and 3-mercaptopyruvate sulfurtransferase (3-MPST) [3, 4, 7–11]. All of them are cytosolic [12–14], but 3-MPST is also localized in the mitochondria [3, 12, 15]. Upon synthesis in different cell compartments such as in the mitochondria, a free form of H_2_S can be released into the cytoplasm or be stored inside the cell as bound sulfane sulfur for subsequent release of H_2_S (Figure 1) [16, 17].

Endogenous H_2_S is a key signaling molecule in humans and other mammals. It has been detected in many organs, and it is involved in the various physiological and pathophysiological processes [12, 18–20]. H_2_S is known to play a role in redox homeostasis and antioxidant responses [21–23], angiogenesis [24–30], vasodilation [31], regulation of synaptic transmission [32], inflammatory responses [33], glucose metabolism [34, 35], ATP production [36], and apoptosis and cell proliferation [23, 31, 37–42]. The role that H_2_S plays in these processes appears to be concentration dependent. The concentration of free H_2_S in plasma could not be measured in a proper way because it is affected by environmental factors such as pH [43], but an initial study utilizing the methylene blue method reported to be between 50 and160 *μ*M in human and rat serum [44]. However, the recent studies are suggesting that the endogenous concentration of H_2_S is much less and is between 10 nM and 3 *μ*M [45, 46]. As H_2_S has a dual effect, at lower concentrations, it has a physiological function in different tissues, whereas at higher concentrations, H_2_S exerts its toxic effects by reversibly blocking of cytochrome C oxidase and inhibiting the electron transport chain in the mitochondria [47–49]. The catabolism of H_2_S occurs mainly in the mitochondria by enzymatic pathways such as oxidoreductases and sulfurtransferase that break it into thiosulfate and sulfate. Moreover, oxidation of H_2_S reduces the FAD prosthetic group, which uses ubiquinone (Q) as an electron acceptor, in electron transport chain which has a role in ATP production (Figure 1) [15, 36, 50–52]. However, under hypoxic conditions, oxidation of H_2_S in the mitochondria reduces, allowing H_2_S to accumulate and function as an oxygen sensor [53, 54]. H_2_S accumulation during hypoxia helps to maintain cell function by upregulating anaerobic metabolic pathways like glycolysis [55] and other cytoprotective pathways [56]. H_2_S also promotes restoration of the tissue oxygen supply by relaxation of vascular smooth muscles (vasodilation) and also stimulation of endothelial cell proliferation and migration (angiogenesis) [24, 57]. Beside the mitochondrial sulfide oxidation [58], H_2_S can be oxidized and catabolized by two other minor pathways [9, 59]. The first pathway is the methylation of H_2_S by thiol S-methyltransferase in the cytosol [60], and the second pathway is an interaction between H_2_S and methemoglobin that leads to the production of sulfhemoglobin and polysulfides, which can be used as a biomarker for plasma H_2_S levels [61, 62].

## 2. H_2_S in Cancer

Several studies have shown that H_2_S and its synthases are associated with the pathophysiology of tumors [20, 49, 63–66]. It has been shown that H_2_S can modulate oxidative stress, interact with free radicals, and activate tumorigenic pathways [39, 61]. Several studies investigated the role and presence of H_2_S in tumors. The expression of H_2_S-producing enzymes (CBS, CSE, and 3-MPST) has been studied in various cancers including liver, colon, ovarian, breast, gastric, lung, oral squamous cell carcinoma, and melanoma [42, 49, 67–74]. However, the role and effect of H_2_S on tumor biology, development, and progression are controversial [75–78]. Previous reviews have adequately summarized that H_2_S can have pro- or anticancerous effects based on the type of tumor and the involved organ [23, 67]. It is reported that endogenous H_2_S can have procancerous effects and help the survival of tumors by stimulating angiogenesis along with promoting cell proliferation, metastasis, and drug resistance [32, 49, 67, 79–81]. The anticancerous effects of exogenous H_2_S administration have been reported for several human cancers [82, 83]. Endogenous H_2_S can be employed as a biomarker for cancer imaging in mice and for differentiating cancer cells [84, 85]. Several pathways, such as inhibition of proliferation, induction of apoptosis, reduction of NF-*κ*B levels, DNA damage, and modification of the cell cycle, are involved in the anticancer activity of H_2_S [27, 29, 82, 86].

Similar to endogenous H_2_S, the effect of exogenous H_2_S treatment also shows a biphasic dose-dependent response on cancer cells as it does in healthy tissues whereby low concentrations of H_2_S exhibit a procancerous effect and high concentrations exert an anticancerous effect [65, 67, 82, 83, 87]. The hypoxic environment of solid tumors [88] leads to a higher level of endogenous H_2_S synthesis [89, 90] and reduces the sulfide detoxification ability of the mitochondria [54, 91], which makes tumors more susceptible to H_2_S toxicity. However, Malagrinò et al. showed that in hypoxic conditions, the activity of the mitochondrial sulfide-oxidizing pathway of quinone oxidoreductase (SQR) adaptively increased and improved the H_2_S detoxification of mitochondria [92].

The direct quantification of H_2_S in tissue samples is a challenge since it has a very short half-life [93]; one study used live fluorescent imaging techniques to visualize the H_2_S in live cells directly [90]. However, in general, the expression level of H_2_S-producing enzymes can be used to indirectly show the correlation between H_2_S and its effects on healthy tissues and tumors [49, 80]. Increased levels of H_2_S and the upregulation of one or more H_2_S-synthesizing enzymes in comparison to healthy tissues have been reported in several tumors [49, 71, 72, 80, 94, 95]. It is also quite interesting that these three enzymes are expressed differently according to the type of cancer [67] and hence lend themselves as potential new targets for therapy.

## 3. H_2_S in Urinary Cancers

Urinary cancers specifically kidney, urothelial, and prostate are relatively common in developed countries. Prostate cancer [96] is the second most commonly diagnosed cancer in men, and urothelial carcinomas (UCs) [97] are the fourth most common tumors both in men and in women. Kidney cancers are highly lethal, and their incidence is increasing incidentally by the common use of diagnostic tools. It is estimated that more than 300,000 new cases of urinary cancers and 33,429 deaths (excluding prostate cancer) will occur in 2019 in the United States [97]. As such, the role of H_2_S and the differential expression of H_2_S-producing enzymes in urinary cancers are of interest, and this review is aimed at summarizing recent evidence on this subject in the context of three common urinary cancers: urothelial cancer, renal cell carcinoma, and prostate cancer.

### 3.1. Urothelial Cancer

Urothelial carcinoma can be located in the lower (bladder and urethra) or the upper (pyelocaliceal cavities and ureter) urinary tract. Bladder tumors account for 90-95% of UCs and are the most common urinary tract malignancy. Sixty percent of upper tract urothelial cancers are invasive at diagnosis compared with 15-25% of bladder tumors [98]. The high recurrence rate and potential of metastasis are two critical characteristics of bladder cancer [99, 100]. Environmental (smoking and exposure to chemical-occupational toxins) and genetic factors all play a role in the etiology of bladder cancer, as does gender since it is more frequent in men older than 65 years of age [101].

Several studies have highlighted the importance of abnormal redox and cellular signaling in the incidence of bladder cancer [102]. Various reports suggest that alterations in H_2_S synthesis pathways may increase the risk of bladder cancer [103, 104], suggesting that the modification of these pathways may lead to the development of novel diagnostic and therapeutic approaches for urological cancers [4].

H_2_S has been detected in bladder homogenates of trout, mice, pigs, rats, and humans [105–109]. In humans, H_2_S is involved in the control of bladder tone homeostasis [110], as it has previously been shown that exogenous H_2_S or its substrate, L-cysteine, could decrease the tone of human and rat bladder strips in a dose-dependent manner [107, 108]. All of the H_2_S-producing enzymes are also found in rat and human bladders, whereas in the mouse, only CSE could be detected [107–109]. The expression of these enzymes in human bladder cancer tissues and cell lines has been investigated. A recent study examined the expression of H_2_S-producing enzymes in human bladder cancer tissues and compared them to healthy ones. They compared 94 human bladder cancer at different stages/grades and 20 human healthy bladder tissues in term of H_2_S content as well as the H_2_S synthases while attempting to find a correlation between the expression of H_2_S-producing enzymes and the malignant progression of bladder cancer. They showed that H_2_S content, as well as the expression of CBS, CSE, and 3-MPST, was higher in bladder cancer than in healthy samples. More interestingly, the enzyme expression of all three enzymes was correlated to different stages of bladder cancer. They suggested that this correlation between the malignancy and the expression of H_2_S enzymes could lead to novel diagnosis and treatment applications [111]. Another recent study also showed, both *in vitro* and *in vivo* models, that apoptosis of bladder cancer cell lines or tissues with cisplatin was enhanced after the inhibition of H_2_S production by propargylglycine (PAG) [23] and was inhibited upon adding the exogenous H_2_S. These authors suggested the activation of the Erk1/2 signaling pathway and the blockage of mitochondrial apoptosis as the possible mechanisms behind their results [112].

Exogenous H_2_S administration has also been shown to affect bladder cancer cell lines. The *in vitro* treatment of the bladder cancer cell line EJ with NaHS enhances cell proliferation and the invasion ability of the cells [113]. Interestingly, these authors also found that the expression of matrix metalloproteinases (MMP) 2 and 9, which are essential for the digestion of collagen IV, was increased in a dose-dependent manner upon the treatment of bladder cancer cells with NaHS. These two enzymes are essential in hydrolyzing the extracellular matrix during the invasion; therefore, H_2_S might be necessary for the invasion of bladder cancer [113]. In addition, nicotinamide phosphoribosyltransferase (Nampt) is the rate-limiting step of nicotinamide adenine dinucleotide synthesis also increased in some cancers [114]. The signal transducer and activator of transcription 3 (Stat3) is one of the cell signaling molecules of the H_2_S, and its activation induces Nampt protein expression via a positive feedback loop. A recent study showed that UC is immunoreactive for the enzymatically active phosphor-Stat3 signal transduction pathway and increased the Nampt and CBS protein expression [115]. Overall, bladder cancer appears to present with higher H_2_S levels in cancer tissue homogenates and increased the expression of H_2_S-producing enzymes, which suggests that H_2_S may be essential for bladder cancer progression and growth, especially in the context of the induction of cell proliferation, inhibition of apoptosis, and facilitation of tissue invasion. Further research is needed to establish consistent expression patterns and other cellular mechanisms for potential diagnostic and therapeutic approaches.

### 3.2. Renal Cell Carcinoma

Renal cell carcinoma (RCC) represents 2-3% of all cancers with the highest incidence in Western countries. The incidence varies globally, with the highest rates in developed countries such as North America and Europe and the lowest rates in Asia and Africa [116]. Over 300,000 men and women are diagnosed with kidney cancer around the world each year, and approximately 150,000 patients will die of the disease [96].

Clear cell renal cell carcinoma (ccRCC), papillary carcinoma, and chromophobe are the common subtypes of RCC [117], although ccRCC accounts for 80% of all RCCs [118]. Because of the lack of early warning signs and the absence of screening tests for people with a higher risk of kidney cancer, more than 30% of patients are at the metastatic stage at the time of diagnosis [119]. Metastatic RCC is highly resistant to systemic chemotherapy and radiation therapy [120, 121].

Inactivation of the Von Hippel-Lindau (VHL) tumor suppressor, which is responsible for the degradation of hypoxia-inducible factor alpha subunits (HIF-1/2*α*) during normoxia, occurs in 90% of ccRCC cases [122, 123]. As a result, HIF-1/2*α* subunits are not degraded under normoxic conditions in RCC cells, and the cells become pseudohypoxic [118]. The Warburg effect, which refers to a shift from mitochondrial respiration to glycolysis and production of lactate [124], enhances tumor growth and metastasis in RCC [125]. Using live cell imaging, Sonke et al. have previously shown that VHL-deficient ccRCC cell lines (769-P and 786-O) have significantly higher H_2_S levels in comparison to ccRCC cells with wild-type VHL (Caki-1). They also showed that the inhibition of H_2_S-producing enzymes by hydroxylamine (HA), which is an inhibitor of CBS and CSE, and PAG, an inhibitor of CSE, significantly decreases the H_2_S levels in VHL-deficient ccRCC cell lines and subsequently inhibits their proliferation and metabolic activity. Moreover, this inhibition of H_2_S synthesis in VHL-deficient ccRCC cell lines results in a twofold reduction in cell survival rate in comparison to untreated cells. Another key finding from this work was that systemic inhibition of H_2_S enzymes by HA administration in xenografted ccRCC in chicken embryos inhibited their vascularization and the subsequent growth of xenografts, which supports the known angiogenic activity of H_2_S [79].

Two more recent studies have also evaluated the expression of H_2_S enzymes in ccRCC. Shackelford et al. compared the expression of CBS in between human benign and Fuhrman grade I-IV ccRCC tissues by using tissue microarray and immunohistochemistry. They showed that CBS expressed weakly in benign tissues and even weaker in Fuhrman grade I ccRCC; however, its expression increased with increasing Fuhrman grades, and CBS expression was the highest in Fuhrman grade IV ccRCC samples [95]. Moreover, the Nmpt expression was correlated with CBS in increasing grade of tumors. Therefore, H_2_S may play a contributory role in the progression of RCC [95]. Breza et al. also investigated the expression of H_2_S-producing enzymes in 21 human ccRCC tissues and compared it to the normal/healthy portion of the same kidney sample using microarray and immunohistochemistry. They found that 66% of ccRCC tissue samples exhibited stable expression of CBS, and the remaining samples showed downregulation. CSE was downregulated in all samples except in three where it was unchanged. The expression of 3-MPST was decreased by 70% of ccRCC samples and remained unchanged in 30% of ccRCC samples [121]. These data suggest that the expression of H_2_S enzymes is heterogeneously regulated in ccRCC. The contradiction between results might be attributed to Shackelford et al. not comparing benign/malignant tissues from the same patient. Breza et al. also showed that, upon induction of apoptosis, the expression of these enzymes was upregulated in the RCC4 cell line (human RCC cell line) and silencing of CBS and CSE expression made the cells resistant to apoptosis [121]. It is possible that endogenous H_2_S induces apoptosis in ccRCC as it was previously reported with exogenous administration [126–130]. The mechanisms behind RCC progression are not well understood, but it is suggested that knocking down of heat shock protein 60 (HSP60) increases the epithelial to mesenchymal transition and enhances invasion and also disturbs the respiratory complex 1 and triggers reactive oxygen molecules and then DNA methylation for further tumorigenesis [131–133]. Tang et al. results supported that suggestion and showed that HSP60 expression is lower in ccRCC tissues compared to pericancerous tissues [134]. The PI3K/AKT pathway is another important pathway in RCC progression, and it is reported that exogenous H_2_S inhibits this pathway, and therefore, exogenous H_2_S could be a novel targeted therapy of RCC [135, 136]. Overall, the expression of H_2_S enzymes could one day become a new tool for establishing prognosis in patients with RCC. However, further studies are necessary to elucidate the exact role of H_2_S in RCC and to explain the contradictions between different studies.

### 3.3. Prostate Cancer

Prostate cancer (PCa) is the second most common cancer in men, with an estimated 1.1 million new cases worldwide in 2012, accounting for 15% of all cancers diagnosed. The incidence of PCa varies widely between different geographical areas, highest in developed countries, mainly due to the use of prostate-specific antigen (PSA) testing and the aging of the population [96]. Surgery, radiotherapy, and androgen deprivation therapies are the primary treatment modalities that are effective, especially in the early stages of the disease [137]. Although a physical exam and the serum PSA test are commonly used to screen and detect for prostate cancer; their utility is ineffective in diagnosing early stages of prostate cancer.

The relationship between H_2_S activity and prostate cancer has been reviewed previously [138]. The expression of H_2_S-producing enzymes was compared between cancerous and healthy prostate tissues [108, 139]. Endogenous H_2_S and all three enzymes (CBS, CSE, and 3-MPST) have been demonstrated in healthy and prostate cancer. CSE has been shown to have a higher expression in the smooth muscle layer of the prostate cancer samples [94]. However, in another study, they could not detect the expression of 3-MPST in both normal and cancerous prostate tissues, but they showed that CSE was significantly downregulated in prostate cancer, whereas CBS was not changed in each sample. This study also showed that antiandrogen-resistant prostate cancer cells express less CSE and have lower H_2_S content in comparison to the parental cell line [140].

Moreover, new evidence suggests that H_2_S-releasing molecules could be effective in the treatment of chemotherapy-resistant prostate cancers [141]. The stromal part of the prostate tissue and the stromal cell line showed average to high CSE expression [139]. In addition, both CBS and CSE are present in mouse prostate cancers, unrelated to androgen dependency, and *in vitro* work showed that CSE is the main contributor to H_2_S production in prostate cancer cell lines (PC-3). The critical role of CSE was confirmed upon finding that aged CSE knockout mice have higher cell proliferation and significantly less H_2_S production in the prostate [142]. Interestingly, the androgen-dependent prostate cell line showed the highest expression of CBS and CSE, and their expression was downregulated upon dihydrotestosterone treatment [139]. These data suggest that CSE may be a potential therapeutic target and diagnostic tool for prostate cancer.

As mentioned earlier, thiosulfate is the stable breakdown product of H_2_S in the mitochondria that can be tracked in the urine. Therefore, the thiosulfate level in urine can be an indicator of exposure to H_2_S or disruption in the breakdown process. Chwatko et al. investigated urinary thiosulfate levels amongst the malignant in comparison to benign prostate hyperplasia (BPH) patients and healthy volunteers. They also found that the urine level of thiosulfate in malignant prostate cancer patients was 50 times higher than the healthy volunteers and five times higher than the BPH patients, and also, there was a positive correlation between the size of the prostate and the urine level of thiosulfate in comparison between the BPH and the control group [143]. In the nude mouse model of human prostate cancer, the plasma concentration of cysteine was significantly decreased after advanced tumor growth [144]. Contrary to these results, five years after prostatectomy, cysteine, homocysteine, and cystathionine were found to be higher in the urine of recurrent prostate cancer patients in comparison to recurrence-free patients [145]. Recent studies showed that methionine catabolism [146], and increased level of cystathionine [147] and sarcosine (N-methylglycine), a by-product of methionine catabolism [148], in urine correlated with prostate cancer stage. In addition, recent data suggest that neuroendocrine-like differentiation of prostate cancer (LNCaP) cells contributes to the androgen-independent growth [149, 150]. The expression and activity of CSE and CBS, in LNCaP cell, are much more than those in healthy prostatic epithelial cells [139]. The H_2_S donors, NaHS and Na_2_S, further enhance the upregulated calcium channels in the LNCaP cells [151]. Overall, it appears that cysteine, homocysteine, cystathionine, and sarcosine could all potentially be biomarkers for prostate cancer.

## 4. Conclusion

Despite significant research efforts in recent years, the role of H_2_S in the context of cancer pathophysiology remains controversial (Table 1). Several studies have partially elucidated the vital role of H_2_S activity, which plays a different role in urological malignancies (Figure 1). Interestingly, the expression patterns of H_2_S-producing enzymes appear to be contradictory, depending upon the subtype of cancer, which was evaluated and in fact, may be tissue dependent. However, these studies, as mentioned earlier, lay the groundwork for future work that may lead to the development of new diagnostic tools for detecting urinary cancers in earlier stages. Moreover, pharmacological modulation of H_2_S synthetic pathways and exogenous administration of donor molecules may one day provide us with additional therapeutic avenues in treating patients with urological malignancies.

## Figures and Tables

**Table 1:** Summary of H_2_S and its producing enzymes in three common urinary cancers.

	H_2_S highlights
Urothelial carcinoma	(i) Expressions of H_2_S and its synthases are higher in cancer tissue [111]. (ii) H_2_S protects the bladder cancer against apoptosis [112]. (iii) H_2_S increases the cell proliferation, invasion, and metastasis of bladder cancer [113].

Renal cell carcinoma	(i) Enhanced expression of H_2_S in ccRCC due to VHL deficiency improves the survival, growth, and metastasis [79]. (ii) Controversial reports about the comparative expression of H_2_S enzymes [95, 121]. (iii) H_2_S contributes to the induction of apoptosis in RCC [121].

Prostate cancer	(i) H_2_S enzymes are expressed in the prostate [107]. (ii) CSE is the main H_2_S-producing enzyme in the prostate [107, 139, 142]. (iii) CSE is downregulated in prostate cancer [140].
